# A novel gastric ulcer model in rats using filter paper with acetic acid

**DOI:** 10.1371/journal.pone.0319096

**Published:** 2025-04-15

**Authors:** Siyuan Dong, Xudong Wang, Yixin Liu, Lu Qiao, Qiong Xue, Yixin Zhou, Zhao Xu, Qun Chen, Chen Chen, Na Liu, Jinhai Wang

**Affiliations:** 1 Xi’an Jiaotong University Health Science Center, Xi’an Jiaotong University, Xi’an, Shaanxi province, P.R. China; 2 College of Safety Science and Engineering, Xi`an University of Science and Technology, Xi’an, Shaanxi province, P.R. China; 3 Department of Gastroenterology, The Second Affiliated Hospital of Xi’an Jiaotong University, Xi’an, Shaanxi province, P.R. China; 4 College of Chemistry, Xi’an Jiaotong University, Xi’an, Shaanxi province, P.R. China; 5 Institute of Endemic Diseases, Key Laboratory of Trace Elements and Endemic Diseases, National Health and Family Planning Commission of the people’s Republic of China, Xi’an Jiaotong University Health Science Center, Xi’an, Shaanxi, P.R. China; 6 Endocrinology, School of Biomedical Sciences, Faculty of Medicine, University of Queensland, Australia; Lahore College for Women University, PAKISTAN

## Abstract

Gastric ulcer animal models are essential for research, but conventional acetic acid-based methods are limited by technical complexity, prolonged modeling duration, and poor applicability for solid anti-ulcer drug testing. This study described a novel and simplified method for inducing gastric ulcers in rats using filter paper imbued with acetic acid. The method provided consistent ulcer formation with uniform size and shape, allowing for targeted drug efficacy testing. Results indicated that the 75% acetic acid concentration yielded the most clinically relevant gastric ulcer model, completed ulcer forming within a day and healing visible after seven days. In conclusion, this model closely replicates human gastric ulcers and is well-suited for preclinical testing of anti-ulcer drugs.

## 1 Introduction

**Fig 1:**
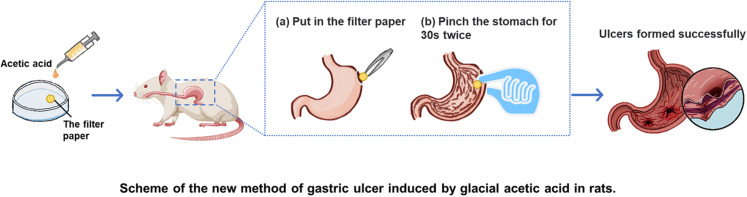
Graphical abstract to summarize the present report.

Peptic ulcer disease (PUD) is a gastrointestinal disorder caused by *Helicobacter pylori* (*H. pylori*) infection and nonsteroid anti-inflammatory drugs [1]. The global prevalence of PUD is 5–10%, with a rate of approximately 7.07% in China, varying between 8.82% in men and 5.4% in women [2]. Gastric ulcers, a subtype of PUD with a global prevalence of 3.15%, are chronic and recurrent, presenting significant clinical challenges [3]. Symptoms include upper abdominal pain and/or discomfort, abdominal distension, acid reflux, nausea, and vomiting. Establishing an easy and good gastric ulcer animal model is crucial for exploring the etiology, pathogenesis, and progression of pathological changes, as well as for defining therapeutic methods and developing new drugs [4]. At present, there are a variety of animal models of gastric ulcer, among which the glacial acetic acid-induced ulcer model is the most widely used one due to its similar occurrence, healing process and pathological morphology to human gastric ulcer. The commonly used glacial acetic acid modeling methods also include filter paper method, glass tube method and injection method [5]. The glass tube method and filter paper method are conducted on serous membrane of the stomach, which poses a high risk of intraperitoneal infection, bleeding, excessive damage, and stomach adhesion to other organs. The injection method, meanwhile, presents potential challenge to precisely control the affected depth of the injection into the stomach wall. As for the efficacy of anti-ulcer medicine, drugs in liquid form maybe intragastrical administrated after successfully establishment of the models with above methods. However, solid drugs are difficult to accurately reach the ulcer site by oral administration. The efficacy for evaluation of solid drugs after successful modeling is therefore difficult and challenging. At present with current modelling methods, it is unsure whether solid drugs are effectively reaching the ulcer site at a given dose. Thus, the comparison of drug efficacy among different candidates may be inaccurate. Therefore, the development of a new animal model that ensured the given dose of drug acting directly on the ulcer site with maximized efficacy and accuracy is urgently required (Fig 1).

An improved rodent model of gastric ulcer, which allows direct drug application to the ulcer site, would be highly valuable for evaluating solid anti-ulcer drugs under endoscopy [6]. However, this method demands advanced surgical skills and extensive training. Its complexity, including the need for a special mold, has limited its widespread adoption in research.

This study aimed to improve the modeling techniques of producing gastric ulcer in rats, which had no physical damage to the gastric wall, and could perform drug test in a self-controlled, dosage-controlled, rapid and easy way. It is hopeful to have this technique widely used in the evaluation of anti-ulcer drug efficacy.

## 2 Materials and methods

### 2.1 Chemicals and reagents

The specific information regarding the chemicals and reagents was provided in Table 1. The filter paper was cut into a small sheet with a diameter of 5 mm. The amount of glacial acetic acid adsorbed by the small sheet ranged from 0.01 mL to 0.10 mL. The mold used in this study was a cylindrical plastic object with a diameter of 5 mm and a length of 1 cm containing a hollow center. The 6–0 suture, 4–0 suture, 2–0 suture and needles were used in this study.

**Table 1:** Detailed Information on Chemicals and Reagents.

Chemicals & reagents	Manufacturer	Batch Number
Sodium chloride injection (NaCl)	Sichuan Kelun Pharmaceutical Co., Ltd.	A22061604
Glucose injection (10%)	China Resources Double-Crane Pharmaceutical Co., Ltd.	220517
Glacial acetic acid	Tianjin Fuyu Fine Chemical Co., Ltd	22020306
Pentobarbital sodium	Sigma-Aldrich (St. Louis, Missouri, USA)	Lbio-005
Filter paper	Bio-Rad Laboratories	10104010

### 2.2 Experimental animals

Thirty-five virgin female Sprague-Dawley (SD) rats weighing between 180 and 220 g were obtained from Animal Center of Xi’an Jiaotong University. The rats were maintained at the room temperature 25 °C± 2 °C and a 12 h light/dark cycle. The animal room was well-ventilated, with humidity controlled between 50% and 70%. The rats had free access to food and distilled water (Milli-Q Direct Water Purification System, USA).

### 2.3 Animals

**Fig 2:**
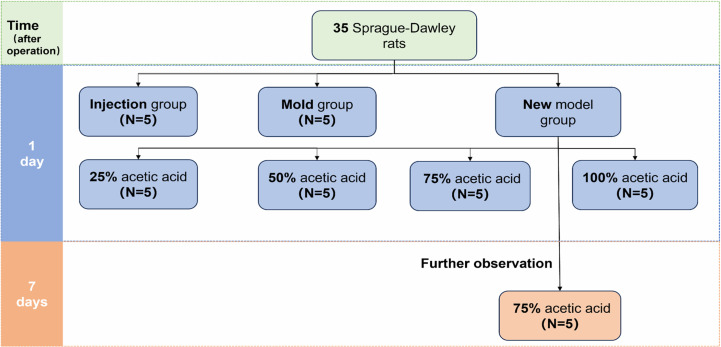
The animal grouping and treatment.

Above mentioned thirty-five SD rats were randomly (random number table) divided into 3 groups, namely, Injection group (5 rats), Mold group (5 rats) and New model group (25 rats). The New model group was then divided into 25% acetic acid group, 50% acetic acid group, 75% acetic acid group, 100% acetic acid group with 5 rats in each group except 10 rats in the 75% acetic acid group. Based on the preliminary experiment, an additional five rats were added to 75% acetic acid group for a prolonged observation of ulcer healing. The animal grouping and treatment were shown in Fig 2 & Table 2. The rats were fasted for 24 h before operation, with free access to water. 1% pentobarbital sodium was injected intraperitoneally at a dose of 30 mL/kg body weight (BW) in rats. The rats were then given skin preparation and fixed supine. The abdominal skin of the rats was disinfected with iodophor and covered with sterile towel. A 2–3 cm longitudinal incision was made from the xiphoid process along the median line of the rat abdomen to open the abdominal cavity and to isolate the rat stomach for further treatment.

**Table 2:** Experimental Groups and Procedures for Gastric Ulcer Induction.

Groups	Procedures
Injection group	Micro-syringe injection Acid delivered between muscular and serous layers
Mold group	Mold placement in stomach Contact with anterior and posterior walls Acid applied for 90 seconds Gastric cavity rinsed and gastric wall sutured
New model group	Acetic acid-soaked filter paper Contact with anterior and posterior walls Applied for 30 seconds twice with 1-minute interval Gastric cavity rinsed and gastric wall sutured

A total of 35 Sprague-Dawley (SD) rats were randomly divided into three groups: the New Model group (25 rats), the Mold group (5 rats), and the Injection group (5 rats). The New Model group consisted of four subgroups: 25% acetic acid (5 rats), 50% acetic acid (5 rats), 75% acetic acid (5 rats), and 100% acetic acid (5 rats). After the procedure, 5 rats from each group were sacrificed after 1 day. Additionally, based on preliminary experiments, five more rats were added to the 75% acetic acid subgroup for extended observation of ulcer healing, being sacrificed 7 days post-operation.

### 2.4 Gastric ulcer induced by the injection

In the Injection group, a 34 G 1.5 mm needle was connected to a micro-syringe, and 0.03 mL 75% acetic acid was injected into the anterior and posterior walls of stomach, respectively. The depth of injection was designed to inject acid between the muscular layer and the serous layer of gastric wall [7].

### 2.5 Gastric ulcer induced by the mold

In the Mold group, 5 mm incision was made at the fundus region along the greater curvature of stomach. The mold was placed into the stomach. The anterior and posterior wall of stomach was covered by the mold placed by hand or toothless forceps, and 0.2 mL 75% acetic acid was injected into the mold. The glacial acetic acid in the mold was then removed after 90-sec exposure, and the stomach cavity was rinsed with sodium chloride injection solution and the stomach wall was sutured [6].

### 2.6 Gastric ulcer induced by the new method

**Fig 3:**
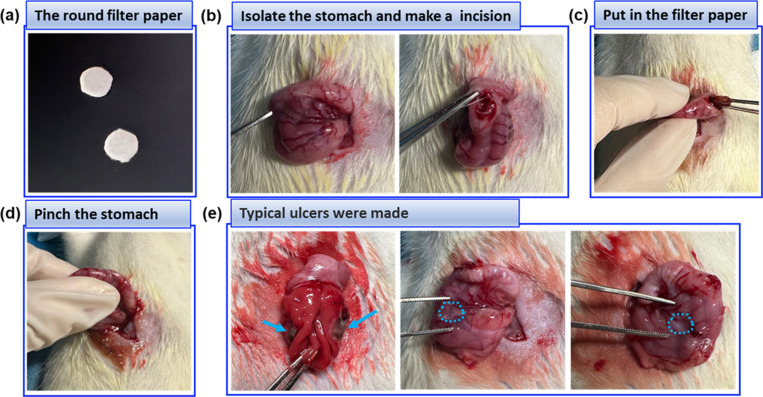
The procedure of the New method in details. (a). The round filter paper with a diameter of 5 mm; (b). The rat stomach was isolated after anesthesia and 2 mm incision was made in the fundus of the stomach; (c). The filter paper absorbed acetic acid was put into the stomach; (d). The anterior and posterior walls of the stomach were pinched by fingers and for up to 30 sec, and repeated twice; (e). The filter paper removal and typical “kissing ulcers” were formed. The abdominal wall was sutured layer by layer after the operation.

In the New model group, the surgical and treatment procedure was shown in Fig 3. An incision of about 5 mm was made at the fundus of stomach along the greater curvature. The filter paper that was weighed and soaked in different concentrations of glacial acetic acid (while the amount of glacial acetic acid was 0.1 mL) was put into the stomach. The anterior and posterior wall of stomach was covered by the filter paper held by hand or toothless forceps for 30 sec twice with1 min interval. The filter paper was then removed, and the stomach wall was sutured with sodium chloride injection after two repetitions.

### 2.7 Post-operation care of rats

After operation, the rats were kept in individual single cages. The standard postoperative procedure was designed to control pain and infection. The rats lied on a heat preservation pad after the operation. 2% ceftiofur sodium at a dose of 3 mg/kg BW was injected intra-bitonally and the tetracaine hydrochloride gel (Zhen’ao Jinyinhua Pharmaceutical Co. Ltd.) was applied to the surgical site. The surgical site was cleaned and redressed daily to prevent infection and to monitor for suture site dehiscence. The rats were fasted for 2 days after the operation, with free access to 5% glucose saline and distilled water. Normal feeding began on the third day after the operation. Daily observations were made and recorded for food intake, water consumption, excretion, and general condition. The experiment was immediately terminated, and euthanasia was performed if the rats exhibited any signs of respiratory distress, diarrhea, urinary incontinence, inability to eat or drink, persistent seizures, abnormal weight gain or loss, or infection and suppuration at the surgical site. Euthanasia was performed using an overdose of pentobarbital sodium, supplemented by bilateral thoracotomy as an auxiliary physical method.

### 2.8 Confirmation of gastric ulcer

Five rats in each group were sacrificed one day after the operation. Based on preliminary results, the 75% acetic acid group exhibited typical gastric ulcer morphology both microscopically and macroscopically, and the general condition of rats was relatively stable. Therefore, the 75% acetic acid group was selected for long-term observation. An additional five rats from the 75% acetic acid group were sacrificed seven days after the operation (Fig 2). The stomach was extracted and rinsed with sodium chloride solution. Subsequently, the stomach was cut open along the large-curved side of stomach. The morphological changes of the gastric mucosa and the ulcers were observed macroscopically. Digital photographs were taken using a digital camera, and the ulcer area was measured using the Image J 2.9.0 image analysis system.

### 2.9 Histological evaluation of gastric mucosa in rats

Gastric mucosa sample was excised from each rat stomach for histological examination in this study. Tissue samples of approximately 0.5 cm × 1 cm were excised from the ulcer margin of the stomach that developed gastric ulcers, as well as from the corresponding location in the stomach without gastric ulcers. The samples were fixed in 4% paraformaldehyde and embedded in paraffin [8]. The paraffin sections were cut at a thickness of 5 µm and stained with hematoxylin and eosin. The extent of inflammatory cell infiltration and mucosal injury was scrutinized under microscope and quantified using the Image J 2.9.0 image analysis system [9].

### 2.10 Statistical analysis

In this experiment, the parameters were measured more than 3 repeats in each group. SPSS 23.0 software (SPSS Inc., Chicago, IL, USA) was used for statistical analysis. All results are presented as mean ± SEM (Standard Error of Mean). The normality of distribution was assessed by the Shapiro-Wilk test. For the examination of homogeneity of variance and the comparison of data among multiple groups, one-way analysis of variance (ANOVA) was employed, followed by Tukey’s post hoc test. Student-t test or Mann-Whitney U was used to analyze the significance in the paired comparison for evaluation of treatment between the anterior and posterior walls in the New model group of each rat. *P* < 0.05 was considered statistically significant. No control group was designed because the focus was to establish a new and better gastric ulcer model in rats in comparison to other established rat models.

### 2.11 Animal experimental ethics statement

This study was performed in strict accordance with the recommendations in the Guide for the Care and Use of Laboratory Animals of the National Institutes of Health. The protocol was approved by the Institutional Animal Care and Use Committee in Xi’an Jiaotong University (approval No. 2022–1295). All experimentalists received prior training at the Laboratory Animal Center of Xi’an Jiaotong University. All methods were reported in accordance with ARRIVE guidelines. All surgery was performed under sodium pentobarbital anesthesia, and every effort was made to minimize suffering.

## 3 Results

### 3.1 The general condition of rats in each group after surgery

All rats survived after surgery. There was no significant difference of excretion and mental state of rats among Injection group, the New model group including 25% acetic acid group, 50% acetic acid group and 75% acetic acid group before and after experimental surgery. The mental states of rats in the Mold group and the New model group of 100% acetic acid group were significantly deteriorated after surgery; showing arched back and hair erection, slow movement, less drinking, etc. The condition of rats from up to 75% acetic acid group was the same as that of the normal rats 7 days after operation.

### 3.2 The macroscopical comparation of ulcers among the new model, injection and mold groups

**Fig 4:**
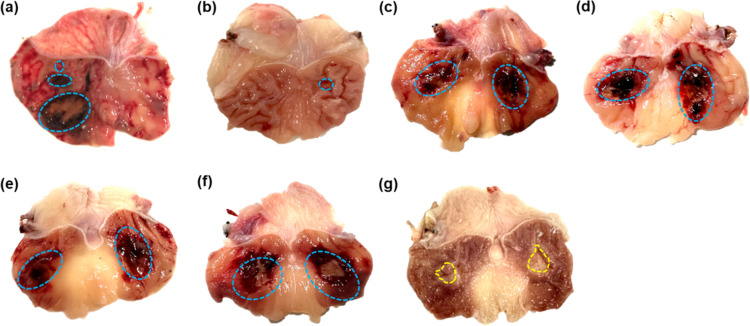
Ulcers were observed macroscopically after surgery. (a). In the Injection group, multiple mucosal defects (blue circles) were found in the gastric wall 1 day after operation, but the depth of the lesions was different, and no ulcer was formed in the symmetrical position. (b). In the Mold group, only a round mucosal defect (blue circle) was observed on the posterior wall 1 day after operation, with no obvious lesions and mucosal edema on the anterior wall, and no obvious signs of congestion and bleeding. (c). In 25% acetic acid group, mucosal hemorrhage, and edema in the symmetrical position of the gastric wall (blue circle) were observed. (d). In 50% acetic acid group, ulcer formation was observed in the symmetrical position of the gastric wall (blue circle), with clear mucosal lesions and peripheral mucosal edema. (e). In 75% acetic acid group, ulcer formation in the gastric wall at the symmetrical position (blue circle) was obvious, and the size was identical with surrounding mucosa congestion and edema. (f). In 100% acetic acid group, ulcer formation was obviously observed in the gastric wall in the same position (arrow) while mucosa was clearly congestion and edema. (g). 7 days after operation in 75% acetic acid group, the ulcer scar was formed in the symmetrical position of the stomach (yellow circles), and the surrounding bulge formed an ulcer spout, which was covered by white moss.

On the first day after operation, the rats were observed and sacrificed. In the Injection group, the abdominal cavity adhesion was serious; the round protrusion was visible on the serous surface of the stomach; and multiple mucosal defects in the anterior wall of the stomach were visible with different sizes and colors. The largest ulcer was black in color, with an area of 0.8094±0.0780 cm^2^, while the rest ulcers were mostly red, with an average area of 0.1818±0.0698 cm^2^. The surrounding mucosa was hyperemia and edema. No obvious ulcers were observed on the posterior wall, possibly due to different injection depths (Fig 4a). In the Mold group, there was no obvious abdominal adhesion. The gastric wall was opened and examined, where only round mucosal defect was observed in the posterior wall of stomach with peripheral mucosal edema. No obvious lesions were found in the anterior wall of stomach, and the ulcer area was 0.0650±0.0036 cm^2^ (Fig 4b).

In the New model group, except for the 100% acetic acid group, the remaining surgical site wounds healed well, and there was no obvious abdominal adhesion or seroperitoneum. In 25% acetic acid group, round or flake mucosal injury was observed in the symmetrical position of the stomach, covered with blood-colored moss, and surrounded by mucosal hyperemia and edema (Fig 4c). In 50% acetic acid group, there were obvious mucosal defects in the symmetrical position in the stomach, and the ulcers were mostly oval and covered by blood-colored moss (Fig 4d). In 75% acetic acid group, kissing ulcers were formed with clear boundaries, and the shape was more regular than that in 50% acetic acid group. The ulcers were mostly round or oval, flat at the bottom, with brown mucosa, partly covered by blood-colored moss, with peripheral mucosa hyperemia and edema (Fig 4e). In the 100% acetic acid group, obvious lesions were observed in the symmetrical position of the stomach; the ulcer surface was red and swollen and in a round or oval shape; the intermediate mucosa was obviously defective, colored brown and slightly white; and the peripheral mucosa was obviously congested with bleeding and significant edema (Fig 4f). Seven days after operation in 75% acetic acid group, the ulcer scar was formed in the symmetrical position of stomach, and the surrounding bulge formed an ulcer spout, which was covered by white moss (Fig 4g).

### 3.3 The comparation of ulcers made by different concentrations of acetic acid

The amount of acetic acid and mean ulcer area in the New model group were shown in Table 3. The ulcer areas of the New model groups were shown in Fig 5. The mean ulcer areas of 50% acetic acid group, 75% acetic acid group and 100% acetic acid group were proportionally increased in comparison with that of 25% acetic acid group (Fig 5a, *P*<0.0001). The mean ulcer area of 75% acetic acid group or 100% acetic acid group was significantly increased from that of 50% acetic acid group (*P*=0.0206<0.05 compared with 75% acetic acid group, *P*=0.0001<0.001 compared with 100% acetic acid group). There was no significant difference in the mean ulcer area between 75% acetic acid group and 100% acetic acid group (*P*=0.3287). There was no significant difference in the mean ulcer area of anterior and posterior wall of the stomach within each group (Fig 5b, *P*=0.6344 for 25% acetic acid group, *P*=0.6652 for 50% acetic acid group. *P*=0.9003 for 75% acetic acid group, *P*=0.9729 for 100% acetic acid group).

**Table 3:** The amount of glacial acetic acid and mean ulcer area for new method.

Group	Number	The amount of glacial acetic acid/mL	Mean ulcer area/cm^2^
25% acetic acid group	5	0.0557±0.0042	0.3350±0.0467
50% acetic acid group	5	0.0508±0.0011	0.5905±0.0790^****^
75% acetic acid group	10	0.0572±0.0058	0.7139±0.0727^****^^,^^#^
100% acetic acid group	5	0.0523±0.0014	0.7790±0. 1021^****^^,^^###^

*Note*:

**** *P*<0.0001, indicated a very significant difference in the mean ulcer area when compared with that in the 25% acetic acid group.

# *P*<0.05,

### *P*<0.001, indicates a significant difference in the mean ulcer area when compared with the 50% acetic acid group.

**Fig 5:**
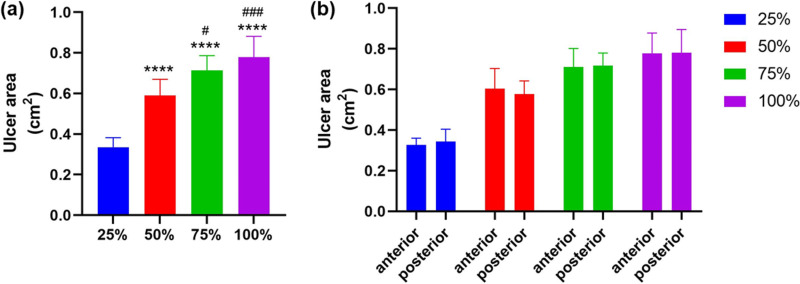
The results of ulcer size determination by the new method ( **S1 Table****).** (a). The calculation results of ulcer area induced by different concentration of acetic acid. (b). The calculation results of similar ulcer sizes between the anterior and posterior gastric wall. ****P<0.0001 (compared to 25% acetic acid group); ^#^*P*<0.05, ^###^*P*<0.001 (compared with 50% acetic acid group).

### 3.4 Ulcer observed by optical microscope

**Fig 6:**
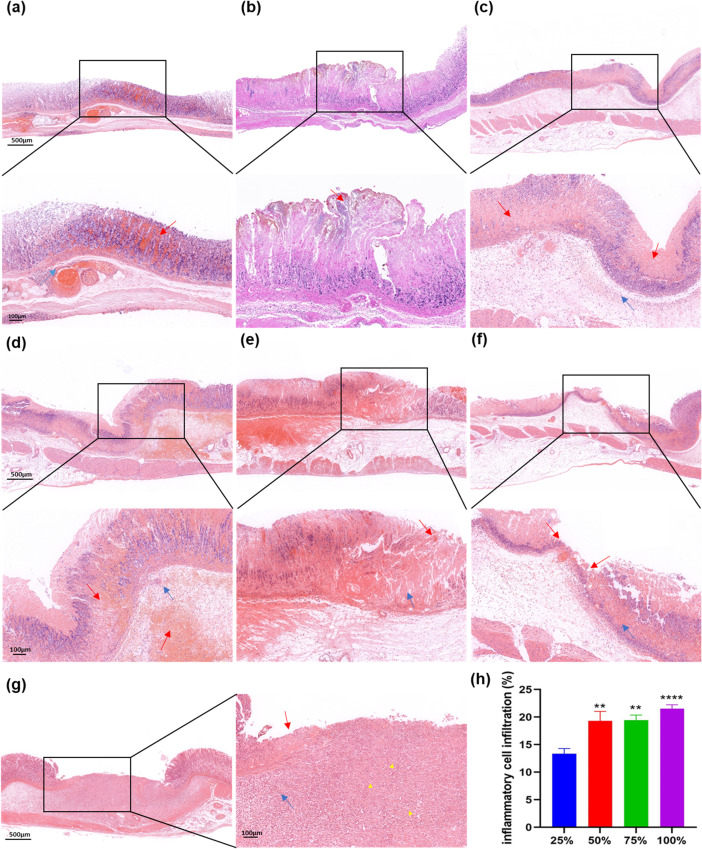
Ulcer formation observed using different methods under the microscope (4x and 10 **×).** (a). In the Injection group under the microscope, the structure of mucosal glands at the largest size ulcer disappeared (blue arrow); the mucosal hyperemia and edema were obvious (red arrow); and some mucosal epithelium was missing. (b). No obvious ulcers were observed in the Mold group, the mucosa epithelium was intact, and the glandular structure was arranged neatly. Only a small amount of mucosal necrosis at the top (red arrow). (c). Mucosal epithelial cell bleeding (red arrow) and inflammatory cell infiltration (blue arrow) were observed in the 25% acetic acid group 1 day after surgery, without mucosal loss. (d). The massive bleeding in the mucosa and submucosa of 50% acetic acid group was observed (red arrow). The infiltration of local inflammatory cells (blue arrow), and partial disappearance of mucosal glandular structure were also observed. (e). The mucosal deletion was observed in 75% acetic acid group (red arrow). The surface was covered with necrotic tissue; mucosal glandular structure disappeared (blue arrow); and the infiltration of many inflammatory cells was observed. (f). In the 100% acetic acid group, the mucosal structure was significantly damaged (red arrow); the surface was covered with necrotic tissue; and the mucosal glandular interstitial was hyperemia and edema surrounding the ulcer (blue arrow). (g). 75% acetic acid group 7 days after surgery, there were significant loss of mucosa at the ulcer, with overlay of necrotic material (red arrow), granulation tissue hyperplasia under the ulcer (yellow triangle), inflammatory cell infiltration (blue arrow), and surrounding mucosa in presence of hyperemia and edema. (h). The degree of inflammatory cell infiltration induced by different concentration of acetic acid (S2 Table). **P<0.01, ****P<0.0001 (compared to 25% acetic acid group). 4x:500 μM, 10x:100 μM.

The gastric tissue of the lesion site was collected. Paraffin-embedded sections of the tissue were observed under light microscope after HE stains. The most obvious lesion in the Injection group was a partial loss of mucosal epithelium. Bleeding on the ulcerative surface was significant, accompanied by inflammatory cell infiltration. The mucosal epithelium structure was intact in the remaining lesions, and some mucosal epithelium had a small amount of bleeding (Fig 6a). One day after surgery, no obvious ulcer was observed in the Mold group; the gastric mucosa epithelium was intact; the cells were arranged neatly; and there was a small amount of inflammatory cell infiltration in the affected mucosal layer (Fig 6b). In 25% acetic acid group, mucosal epithelial bleeding was obvious; cell edema was clearly observed; and many inflammatory cells were infiltrated in the submucosa; but no obvious mucosal loss was detected (Fig 6c**).** In the 50% acetic acid group, there was obvious bleeding in the mucosa and submucosa; mucosal glandular structure disappeared; and a large number of inflammatory cells were infiltrated the submucosa (Fig 6d). In 75% acetic acid group, the ulcer site was characterized by a loss of mucosal epithelium; the surface of ulcer was covered by necrotic tissues; the peripheral mucosal glandular structure disappeared; and the infiltration of a large number of inflammatory cells was observed in the mucosa and submucosa (Fig 6e). In the 100% acetic acid group, there was significant damage to the mucosal structure at the ulcer site, with a loss of mucosal epithelium and mucosal glands, a massive bleeding on the surface and submucosa of the ulcer, a significant inflammatory cell infiltration, and a congestion and edema in the glandular interstitial of the mucosa surrounding the ulcer (Fig 6f). Further quantitative analysis revealed that compared with 25% acetic acid group, the degrees of inflammatory cell infiltration in 50%, 75% and 100% acetic acid group were significantly increased (50% group: *P* = 0.0031 < 0.01, 75% group: *P* = 0.0012 < 0.01, 100% group: *P* < 0.0001). However, there was no significant difference in the degree of inflammatory cell infiltration among the 50%, 75%, and 100% acetic acid groups (Fig 6h). Seven days after surgery, compared to the day 1, the mucosa epithelium at the ulcer in the 75% acetic acid group was obviously missing; the glands were disappeared; the necrotic tissue was less at the position of ulcer; the granulation tissue hyperplasia was visible under the ulcer; there was less inflammatory cell infiltration; and the mucosal glandular interstitial around the ulcer was congested with edema (Fig 6g).

## 4 Discussion

Gastric ulcer is a globally prevalent disease. Bleeding, perforation, stomach outlet obstruction and other common complications often bring great pain to patients [10]. To deeply understand and explore the pathogenesis and to have a more effective treatment of gastric ulcer, the careful animal experiments are required [11]. Currently, commonly used ulcer models are divided into stress induced model, non-stress induced model, exercise stress model, drug induced model, ethanol induced model, acetic acid and glacial acetic acid induced model, and pyloric ligation induced model [12–18]. Among them, gastric ulcer model induced by glacial acetic acid and/or acetic acid is the most widely used one because of its similar pathological characteristics and healing process to those in human chronic ulcer [19]. The common methods of glacial acetic acid induced gastric ulcer model are as follows, with serious limitations.

(1) Injection method: Experimental rodent was fixed supine, and the abdominal wall was cut open to the stomach. An appropriate amount of glacial acetic acid was injected into the space between the muscular layer of the gastric wall and the serous layer with a syringe [20]. During and after the injection, it is essential to apply pressure to the needle to prevent the leaking of acetic acid into the abdominal cavity [21]. As such, this method is technically complex, requires a prolonged modeling time, and carries a risk of physical damage on the injection site. Furthermore, localized diffusion of acetic acid may damage other close-by intra-abdominal organs, particularly the liver [22]. The diffusion of different injection sites is highly variable, and the depth and size of created ulcers are very difficult to be quantified. The evaluation of drug treatment of ulcers is equally difficult.
Due to the relatively thin stomach wall in rat, the depth of injection is hard to control, and the similar depth of injections cannot be guaranteed [23]. In this study, following the injection into the serous surface, the rats exhibited pronounced adhesion in the abdominal cavity, with no apparent formation of ulcers observed in the stomach. Tong et al. conducted a detailed analysis of the acetic acid gastric ulcer model and revealed that the ulcer area in the acetic acid injection group was not significantly different compared to that in the normal control-injection without acetic acid group [5]. Furthermore, needles penetrated the stomach wall, causing perforation and subsequent mortality of a number of rats [5]. The injection methods caused severe organ adhesions, differing from the clinical characteristics of human gastric ulcer [7]. Therefore, the surgical accuracy of the injection method is challenging, and the strong experimental skill is needed to ensure the success.
(2) Glass tube method: After the rats were anesthetized and the stomach was isolated, the glass tube was pressed vertically at the serous surface of the gastric antrum, glacial acetic acid was injected into the glass tube and removed after 1 minute [24]. The stomach was then sutured. Usually 3–5 days after operation, the ulcer was formed. Similar to the injection method, this approach had a propensity to induce adhesion among abdominal organs, particularly to the liver [7]. The glass tube method did not ensure that the drug was exactly applied to the ulcer, thus the comparison of drug efficacy was inaccurate. This method may cause leakage of acetic acid, leading to unspecific damage to the abdominal organs.
(3) Filter paper method: After the stomach was isolated in rat, the filter paper impregnated with glacial acetic acid was attached to the serous surface of the stomach wall for a period of time, and then the filter paper was removed. The abdominal wall was washed with normal saline and then stitched [5]. At the fourth day post-surgery, the regions of mucosa not directly exposed to acetic acid also exhibited pathological changes in a mouse Filter-paper experiment [24]. The filter paper attachment method also resulted in organ adhesions in rats, with significant trauma to the spleen [5].

It is difficult to quantify the drug dose in above ulcer modeling due to the treatment of the gastric serosa surface in the methods (2) and (3). The glacial acetic acid may flow out along the gastric wall and cause glacial acetic acid corrosion in the other parts of gastric wall, even in the abdominal cavity or other organs, which affects the survival rate of rodents. In addition, the methods (2) and (3) are difficult to form a pair of similar ulcers for self-controlled drug test. In addition, the time to form a model is relatively long. The glass tube method may have some physical damage to the gastric wall too. The New model method reported here was simple and easy. In addition, this new method guaranteed that the drug acted directly on the ulcer without the acetic acid leakage.

For drug efficacy evaluation study, the above three methods have certain limitations to test non-liquid drugs and drugs used with endoscopy. Although liquid drugs may be tested by intragastric administration after successful modeling, solid drugs may not be applied to the ulcer sites after intragastric administration, and the drug dosage may not be accurately quantified. Careful self-controlled experiments in same animal may not be able to perform either. The evaluation of drug efficacy has obvious been compromised.

An improved method for the modeling of gastric ulcer in rats was reported recently [6]. After the stomach of the rats was isolated, an “O” mold was used. The mold was placed into the stomach and held with the hand, glacial acetic acid was injected into the mold for 90 sec, and the ulcer was formed in the stomach. However, in actual operation, after injecting glacial acetic acid into the mold, the contact time of the anterior or posterior gastric wall of the stomach was variable. It was also difficult to quantify the dosage of the drug, and the operation was complex requiring surgical skills. Therefore, such model is not widely used yet.

The current study aimed to provide a new model of gastric ulcer in rats, which had advanced drug testing system with self-controlled comparison, dosage-controlled ulcer size and depth, rapid modeling process, and easy to perform. Employed round filter paper was regular in shape and had no physical damage to the stomach wall. The filter paper was cheap and easy to obtain. In addition, due to the adsorptive properties of the filter paper, successful modeling allowed for the application of drug-laden filter paper to the gastric wall. By the measurement of filter paper weight before and after insertion into the gastric wall, the dosage of drug applied to the gastric wall could be calculated. This approach enabled a more precise assessment of therapeutic efficacy of the administered drug. Moreover, after the filter paper was placed inside the stomach, the anterior and posterior gastric walls of the stomach formed a sandwich with the filter paper causing two identical ulcers of same size, shape, and extend in the symmetric positions of the stomach, namely “kissing ulcers”. Such “kissing ulcers” achieve the purpose of experiment with self-controlled comparison.

The overall surgery of this New model was much simpler than other models, and the experiment was highly repeatable. In addition, the modeling time was as short as only 1 day. After the operation, the gastric ulcers were obvious, and the size and depth were identical among the same dose group. In the pre-experiment, the New model group (75% acetic acid group and 100% acetic acid group) displayed typical ulcer formation 1 day after surgery, with ulcer scar formation observed 7 days post-surgery. While the injection group and the low dose New model groups (25% acetic acid group and 50% acetic acid group) exhibited no typical ulcer formation at 1 day after surgery. Based on the experimental findings, the ulcer observed in the 75% acetic acid group exhibited the most typical characteristics of human gastric ulcer, and the overall condition of experimental rats was relatively favorable for the recovery test. Consequently, an additional five rats were added for a prolonged observation of ulcer healing process at 7 days post-surgery.

After 7 days, the gastric wall was dissected with obvious ulcer scar formation, which was convenient for the calculation of ulcer area and evaluation of drug effect. In addition, this study explored the variable concentrations of glacial acetic acid. The rats using 25%, 50% and 75% acetic acid concentrations had a very good post-operation recovery with very high survival rate. The 25% acetic acid caused only mucosal congestion and edema, without ulcer formation. Although ulcer-like lesions were observed macroscopically in the stomach of 50% acetic acid group, no obvious mucosal defects were observed under the light microscope. Only 75% acetic acid showed clear ulceration both macroscopically and under light microscope. Ulcers were typical when the concentration was 100%, with poor recovery after surgery. Therefore, the 75% acetic acid was ultimately recommended.

This New modeling method provides a versatile platform for evaluating both liquid and solid drugs. The model allows drugs to act directly on the ulcer site, enabling more accurate and efficient evaluations of therapeutic efficacy. For solid drugs specifically, the paired “kissing ulcers” in the anterior and posterior gastric walls allow for one ulcer to be treated while the other used as a self-control. This eliminates inter-animal variability and improves the reliability of experimental results. In addition, this model mimics the clinical scenario of endoscopic drug delivery, offering a preclinical platform to deliver drugs directly on to the gastric ulcer sites under endoscopy. This application provides significant clinical relevance, as it fills the gap between preclinical studies and clinical drug delivery methods.

This study has however certain limitations that should be acknowledged. The model was only validated in rats, and its applicability to other species remained to be explored. Additionally, further research may be needed to determine the suitability for testing drugs with different physicochemical properties, particularly those requiring sustained release or complex formulations. Future studies should also be performed to investigate the long-term effects of repeated drug administration simulating chronic treatment scenarios.

## 5 Conclusions

In conclusion, this New acetic acid-induced gastric ulcer model in rats represents herein a protocol with a short modeling duration, cost-effective process, defined ulcer lesion site, and reliable internal controlled ulcer pairs. This model has advantageous in evaluating drug efficacy, and improving limitations of previous acetic acid-induced gastric ulcer models. Specifically, this model allows tested drugs to act directly on the ulcer site. Compared to traditional intragastric administration, this model enables more accurate assessment of therapeutic effects, particularly for solid drugs. Moreover, it simulates the efficacy evaluation of drugs delivered directly via endoscopic methods. Thus, it represents a straightforward, and highly efficient novel gastric ulcer animal model that may advance preclinical research in gastric ulcer treatment.

## Supporting information

S1 Table Descriptive statistics of Fig 5. (XLSX)

S2 Table Descriptive statistics of Fig 6h. (XLSX)
